# EU Fiscal Rules: A Look Back and the Way Forward

**DOI:** 10.1007/s10272-022-1020-2

**Published:** 2022-02-12

**Authors:** Klaus Regling

**Affiliations:** European Stability Mechanism, 6a Circuit de La Foire Internationale, 1347 Luxembourg, Luxembourg

**Keywords:** E62, H6

## Abstract

The decline of “natural real interest rates” during the last three decades is a common phenomenon in all advanced economies and can be explained by a number of structural factors: the downward trend in potential growth; population ageing, which has led to higher savings rates in many countries; and the rising inequality in the distribution of wealth, which also increases the average savings rate.

Safeguarding fiscal sustainability has been a key element of our Economic and Monetary Union (EMU) from the very beginning. This reflects the fact that in the EMU, monetary policy is fully centralised while fiscal policy is conducted at the national level. Consequently, it is necessary to coordinate fiscal policies to ensure sound government finances, which in turn are essential for preserving debt sustainability, maintaining trust between the member states and strengthening the confidence of citizens and financial markets in the euro. When the Maastricht Treaty was signed in 1992, it included two reference values that to this day remain at the core of EU fiscal rules: a fiscal cap of 3% of GDP and public debt target of 60%.

These reference values were part of the convergence criteria that govern the entry into the EMU. The Stability and Growth Pact (SGP), signed in June 1997, operationalised the reference values for fiscal coordination after the launch of the EMU in 1999. The Pact specified that member states should aim for a balanced budget in cyclically normal times or have a budgetary balance on average over the cycle, but not exceed the 3% deficit cap. The purpose was to allow countercyclical policies in order to create fiscal space in a recession, when the fiscal deficit could move from balance to a maximum deficit of 3% of GDP. In addition, the SGP introduced an escape clause (“exceptional circumstances”) that allowed larger deficits in case of severe downturns.

After the beginning of the EMU, however, many governments did not use higher revenues in cyclical upturns to reduce deficits and thus did not create sufficient fiscal space. The periods of economic growth in the late 1990s and mid-2000s were not used to create sufficient buffers. Fiscal policy was often procyclical during good times. Consequently, dealing with an economic downturn required procyclical tightening or non-compliance with the rules.

The peer pressure expected to support the SGP was insufficient, as divergent views among EU member states broke the consensus on the need to strictly comply with the rules. A ruling in 2004 by the European Court of Justice effectively allowed EU member states (through the EU Council) to postpone the implementation of measures recommended by the Commission to reduce excessive deficits.

The relatively simple initial structure of the SGP later underwent substantial revisions that increased its complexity. The first such reform, carried out in 2005, introduced the concept of “structural balances” and extended the deadline for correcting excessive deficits.

However, the focus on structural balances presents methodological problems, as it relies on the calculation of output gaps – an indicator that is not observable and subject to frequent *ex post* revisions. Output gaps as estimated by various international institutions yield diverging results, which underlines how difficult it is to agree on reliable figures.

As a result, the use of structural balances to assess compliance with the rules was increasingly questioned after the global financial crisis in 2008-2009, when revisions in output gap estimates were particularly sizeable. Frequent revisions of potential GDP and the output gap have led to scepticism concerning the credibility of rules based on cyclically adjusted variables.

The 2011 revisions, carried out through the “six-pack” legislation, strengthened fiscal surveillance and broadened the scope of economic surveillance to include macroeconomic imbalances. The changes included an expenditure benchmark and an operationalised debt reduction rule, which provided that a country’s debt ratio should decline at an annual rate of one-twentieth of the excess of the debt ratio over the 60% debt-to-GDP target.

In 2015, further changes made fiscal adjustment requirements more flexible. As a result of the increasing degree of sophistication of the SGP – driven by the desire to make the framework more adaptable to countries’ specific circumstances – the rules have become more complex, which has reduced the overall transparency of the framework.

Despite these shortcomings, the Stability and Growth Pact brought benefits during the first two decades of the EMU. Fiscal rules helped to achieve lower fiscal deficits and debt levels in the euro area compared to almost all other advanced economies.

### The fiscal framework in a new macroeconomic environment

The economic landscape has evolved significantly since the SGP's inception and Europe is facing new challenges. The economic and financial context is shaped by new long-term trends, especially with regard to the interest rate environment. The yield on ten-year German government bonds provides a good illustration of that trend: During the 1980s, the average yield amounted to 7.6%. In the 1990s, it fell to 6.6% and from 2010 until the start of the pandemic, it was 1.1%. In other euro area countries, this development has been even more striking, as the EMU led to interest rate convergence at a low level.

The decline of "natural real interest rates" during the last three decades is a common phenomenon in all advanced economies and can be explained by a number of structural factors: the downward trend in potential growth; population ageing, which has led to higher savings rates in many countries; and the rising inequality in the distribution of wealth, which also increases the average savings rate.

These factors are unlikely to change in the foreseeable future, even though interest rates will increase from today's extremely low, pandemic-induced levels, and even as public and private investment will grow to finance the transformation of our economies towards green and digital. On average, interest rates can be expected to remain well below previous levels, significantly lower than 30 years ago when the Maastricht Treaty was negotiated.

The decline in interest rates relative to economic growth, and the expectation that they will remain low for long, expand the capacity of euro area members to service debt. Member states incurred substantial additional debt to mitigate the economic impact of past crises and of the COVID-19 pandemic, but the budgetary costs of indebtedness (i.e. interest payments) have drastically declined in nominal and effective terms and are expected to remain low. The contraction of interest payments compared to the level of debt has been common to all advanced economies around the world, including euro area countries.

Europe's fiscal response to the pandemic crisis, along with decisive European Central Bank (ECB) action, have provided essential support for EU economies during difficult times. The pandemic fiscal support pushed the average euro area debt ratio to almost 100% of GDP in 2020, from around 86% in 2019. Some countries now face significantly higher debt ratios. But the higher debt levels do not necessarily imply higher sustainability risks, as financing costs are lower and are expected to remain low in the foreseeable future.

### Proposal for a new EU fiscal framework

A return to the pre-pandemic EU fiscal framework is not advisable for the reasons mentioned above. The shortcomings of the current framework were known already before the coronavirus pandemic, and that is why the European Commission launched a review of EU economic governance rules in February 2020. Making use of existing flexibilities to circumvent the flaws may not be efficient. The reform of EU fiscal rules should provide a credible and transparent framework supporting growth and investment and, at the same time, encourage a realistic and transparent debt reduction where needed to guarantee debt sustainability for all euro area members.

In my view, a simplified and credible EU fiscal framework could be built around a 3% deficit limit, a higher limit for the debt-to-GDP target, and a combination of an expenditure and a primary balance rule targeting debt reduction in good economic times.

The 3% deficit-to-GDP reference value, which appears broadly acceptable, could be preserved as a limit whose breach would trigger proceedings concerning "gross policy errors" that would normally lead to an excessive deficit procedure. However, the 60% debt ceiling – a useful benchmark reflecting the prevailing financing conditions at the time of the Maastricht negotiations in the 1980s – no longer appears appropriate when looking at the changed macroeconomic context. Forcing all euro area member states to meet such a reference value over a 20-year horizon would require unnecessarily large fiscal surpluses for several countries. Delivering such surpluses would be detrimental to the countries' growth prospects, politically hard to implement and, importantly, not necessary to guarantee debt sustainability.

Raising the debt ceiling should not be misunderstood: It does not mean that countries should continue to spend freely. Maintaining debt sustainability must remain the main objective of the fiscal surveillance framework, and high government debt – in particular if above 100% of GDP – should always be reduced when macroeconomic conditions permit. Depending on national circumstances, countries need to prepare for the additional fiscal costs of ageing and climate change. It is also important to create fiscal space to respond to the next crisis.

An effective way to contain the growth rate of government spending would be the introduction of an expenditure rule, which has been proposed by many observers. For all member states, the growth in public expenditure should not be higher than the trend growth rate. The main advantages of expenditure rules are transparency and accountability, as expenditures are controlled by governments and parliaments and can be monitored easily with very short time lags. In addition to the expenditure rule, countries with debt ratios above the euro area average should follow a realistic debt reduction path determined by a primary balance rule.

Another important consideration for future discussions is the use of debt to finance productive public investment. An appropriate level of public investment will be crucial to respond to the challenges of climate change and digitalisation. Reforming EU fiscal rules in a way that supports and incentivises green and digital investments without jeopardising debt sustainability would be desirable but is not easy to achieve.

Debt-financed public investment increases public debt in the first instance; whether it leads to additional growth, thus reducing sustainability risks over time, requires careful analysis. In any case, a higher debt reference value would also provide more space for financing additional productive investment compared to the existing rules. And other existing coordination mechanisms and policy tools such as the European Semester, the Recovery and Resilience Facility, the EU budget and loans from the European Investment Bank, should also be used to finance the transformation of EU economies. The effective use of funds received from these mechanisms can be strengthened through proper expenditure management at the national level, which could strengthen the quality of budgets more broadly.

## SGP and the EU legal framework

The provisions containing the SGP's 3% and 60% reference values are in Protocol 12 of the EU Treaties, which could be modified by making use of a special legislative procedure that allows amendments to the individual provisions of Protocol 12. These amendments would require unanimity in the Council, as well as a consultation of the ECB and the European Parliament. In my view, this would avoid the necessity to go for a full Treaty revision procedure.

The one-twentieth debt reduction rule is laid down in both EU law (a Council Regulation) and international law (Treaty on Stability, Coordination and Governance in the Economic and Monetary Union, comprising the Fiscal Compact). Arguably, the most feasible way to amend the rule would be to incorporate the Fiscal Compact into EU law.

## Conclusion

Fiscal sustainability today is no less important than 30 years ago when the EMU was established. A credible fiscal framework is needed that takes into account the current macroeconomic context. Returning to a strict debt adjustment requirement based on a target of 60% of GDP would be hard to defend from an economic perspective and would therefore weaken commitment to the rules. The low interest rate environment – expected to persist in the foreseeable future – alleviates sustainability concerns to some extent but future fiscal burdens must be anticipated.

Agreeing on new rules and phasing them in once the economic situation normalises could help guide market expectations and contain potential volatility. Hence, a timely agreement on new rules, and on a timeline and conditions for their implementation, could help stabilise expectations and increase transparency. Credible debt reduction paths for countries with debt ratios above the euro area average could also help ensure favourable market financing conditions. More broadly, an economically credible framework, with a renewed commitment by all member states to implement the revised rules, would be welcomed by financial markets, thus reinforcing today's positive view of markets on Europe and strengthening the international role of the euro.
